# Nucleosome remodeling and deacetylation complex and MBD3 influence mouse embryonic stem cell naïve pluripotency under inhibition of protein kinase C

**DOI:** 10.1038/s41420-022-01131-0

**Published:** 2022-08-01

**Authors:** Yujian Dai, Jialei Sun, Na He, Liyou An, Chunhui Hou, Fuliang Du

**Affiliations:** 1 Jiangsu Key Laboratory for Molecular and Medical Biotechnology, College of Life Sciences, Nanjing Normal University, 210046 Nanjing, China; 2 Harbin Institute of Technology, 150001 Harbin, Heilongjiang China; 3 Xinjiang Key Laboratory of Biological Resources and Genetic Engineering, College of Life Science and Technology, Xinjiang University, 830046 Urumqi, China; 4 Shenzhen Key Laboratory of Gene Regulation and Systems Biology, Department of Biology, School of Life Sciences, Southern University of Science and Technology, 518055 Shenzhen, Guangdong China

**Keywords:** Embryonic stem cells, Self-renewal

## Abstract

The pluripotency of naïve mouse embryonic stem cells (mES) is regulated by multiple signaling pathways, with inhibition of protein kinase C (PKCi) playing a particularly important role in maintaining naïve mES. However, the regulatory function of nucleosome remodeling and deacetylase (NuRD) complex in mES cultured in a PKCi system is unknown. We found that, compared with 2iL-derived mES, PKCi-derived mES showed low mRNA expression of NuRD complex subunits, including *MBD3*, *HDAC1*/*HDAC2*, *MTA1*, and *RbAP46*/*RbAP48*. Western blot showed that PKCi-derived mES expressed lower protein levels of MBD3 and HDAC2 at passage 3, as well as MBD3, HDAC2, and MTA1 at passage 10, indicating that PKCi suppressed NuRD complex expression. Knockdown of *MBD3* increased PKCi-derived mES pluripotency by increasing *NANOG* and *OCT4* expression and colony formation. By contrast, overexpression of MBD3 or removal of PKC inhibitor-induced differentiation of mES, results in reduced *NANOG*, *OCT4*, and *REX1* expression and colony formation, increased differentiation-related gene expression, and differentiation into flat cells. Knockdown of *MBD3* in mES upon PKC inhibitor removal partially reversed cell differentiation. Our results show that the regulatory NuRD complex and its MBD3 subunit influence the naïve pluripotency of mES cultured in a PKCi system.

## Introduction

The nuclear remodeling and deacetylation (NuRD) complex is an abundant and conserved regulator of chromatin structure remodeling and transcriptional repression [1, 2]. The NuRD complex contains several subunits (e.g., methyl-CpG-binding domain protein MBD2/3, histone deacetylase core proteins HDAC1/2, metastasis-associated protein MTA1/2/3, ATP-dependent nucleosome remodeling enzyme CHD3/4, histone-binding/chaperone proteins RbAP46/48, zinc-finger proteins p66α/β, and DOC1), mediates two major biological functions: nucleosome remodeling in chromatin formation and histone deacetylation, resulting in the silencing of gene transcription [3]. NuRD was recently found to modulate chromatin structure at regulatory elements of active transcription sites, thereby regulating gene expression in a finely tuned manner [1].

Embryonic stem cells (ES), first derived by Evans and Kaufman in 1981 [4], possess the characteristics of self-renewal, indefinite proliferation in vitro, multi-lineage differentiation, and germline transmission. Mouse ES (mES) can exist in naïve or primed states [5]. Two different culture systems can maintain mES naïve pluripotency: 2iL and inhibition of the PKC signaling pathway (PKCi). The 2iL system includes leukemia inhibitory factor (LIF), which activates transcription factor signal transducers and activators of transcription 3 (STAT3) and the small-molecule inhibitors PD0325901 and CHIR99021, which in turn inhibit mitogen-activated protein kinase (MAPK) and glycogen synthase kinase 3 (GSK3) pathways, respectively [6, 7]. 2iL-derived mES are frequently used in stem cell studies. The PKCi system also maintains the naïve state of mES and rat ESs [8, 9]. Members of the PKC family are intracellular mediators of several hormones, neurotransmitters, phorbol esters, and tumor promoters that play essential roles in growth regulation, ES self-renewal, cell differentiation, neurotransmission, and cell death [10]. In particular, inhibition of the PKCζ–NF-κB–microRNA-21/microRNA-29 axis is key to maintaining ES self-renewal and naïve pluripotency [8, 9]. However, the role of NuRD subunits in PKCi-derived naïve mES, as well as differences in NuRD complex expression between 2iL- and PKCi-derived mES, are unknown.

Previous studies revealed the mechanisms by which NuRD maintains stem cell pluripotency via the regulation of gene expression related to cell plasticity, self-renewal, and differentiation during development [1, 11]. MBD3, a specific ES NuRD subunit, is essential for early embryogenesis and the development of pluripotent stem cells [12, 13]. As a methylated DNA binding protein that scaffolds other NuRD subunits, MBD3 is crucial for recruiting other subunits and assembling the NuRD complex [14, 15] and modulates transcriptional heterogeneity, and maintains ES lineage commitment by directly regulating the expression of pluripotency genes in ESs [16]. MBD3 is required for commitment to a full spectrum of embryonic lineages [15, 17, 18]. *MBD3*^*−/−*^ ES maintains the expression of pluripotency genes such as *OCT4* and *NANOG* but fails to form stable NuRD complexes, exhibit severe defects in differentiation [12], and shows silencing of *Oct4* expression upon withdrawal of LIF [15]. However, the role of MBD3 in the growth and self-renewal of mES colonies in the PKCi system is unclear. In particular, identifying differences in target gene regulatory loci specifically recognized by MBD3 between 2iL- and PKCi-derived mES will provide a fuller understanding of how different signaling pathways induce naïve mES.

In this study, we examined differences in NuRD complex RNA and protein expression in naïve mES derived in 2iL versus PKCi culture systems. We found that NuRD subunits are present in both types of mES, suggesting that NuRD is necessary for maintaining ES self-renewal and pluripotency. However, the RNA expression of most NuRD subunits, including HDAC2, MBD3, and MTA1, was lower in PKCi-derived mES than in 2iL-derived mES. Furthermore, using overexpression and knockdown of *MBD3*, we demonstrate that NuRD and MBD3 play important roles in PKCi-derived mES self-renewal and naïve pluripotency by modulating the expression of pluripotency and differentiation genes.

## Results

### PKCi downregulated expression of the NuRD complex in mES

**Fig. 1:**
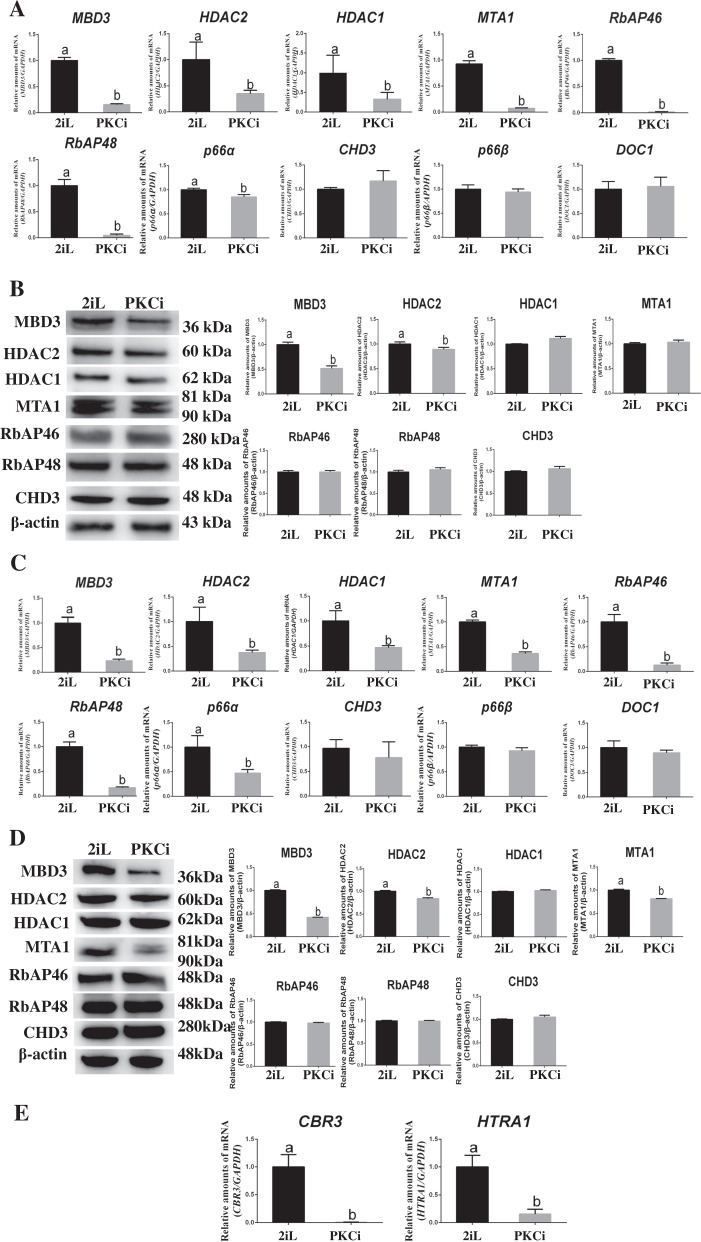
PKCi downregulated expression of the NuRD complex. **A** qPCR showed that compared with 2iL, PKCi decreased mRNA levels of NuRD complex subunits *MBD3, HDAC2*, *HDAC1*, *MTA1*, *RbAP46*, *RbAP48*, and *p66α*, but not of *CHD3, p66β*, and *DOC1*, in mES at passage 3. **B** Protein levels of NuRD complex and β-actin were evaluated by Western blot (left panel). Quantitative density analysis showed that compared with 2iL, PKCi decreased protein levels of MBD3 and HDAC2, but not other subunits (right panel). **C** qPCR showed that compared with 2iL, PKCi decreased mRNA levels of *MBD3, HDAC2*, *HDAC1*, *MTA1*, *RbAP46*, *RbAP48*, and *p66α*, but not of *CHD3, p666β*, and *DOC1*, in mES at passage 10. **D** Protein levels of NuRD complex and β-actin were evaluated by Western blot (left panel). Quantitative density analysis showed that compared with 2iL, PKCi decreased protein levels of MBD3, HDAC2, and further MTA1, but not of other subunits (right panel). **E** qPCR showed that compared with 2iL, PKCi significantly decreased the mRNA levels of NuRD target genes including *CBR3* and *HTRA1* in mES at passage 3. Data were shown as mean ± SD (*n* = 3). The letters a and b indicated significant differences among groups (*P* < 0.05).

To investigate how the PKCi culture system affects the expression of NuRD complex components, we first measured the mRNA expression of the NuRD complex in PKCi-derived mES at passage 3. Quantitative polymerase chain reaction (qPCR) showed that compared with 2iL-derived mES, PKCi-derived mES showed lower mRNA levels of *MBD3* (from 1.0 to 0.2), *HDAC2* (from 1.0 to 0.4), *HDAC1* (from 1.0 to 0.3), *MTA1* (from 1.0 to 0.1), *RbAP46* (from 1.0 to 0.02), *RbAP48* (from 1.0 to 0.05), and *p66α* (from 1.0 to 0.8) (*P* < 0.05; Fig. 1A), whereas *CHD3*, *p66β*, and *DOC1* levels were similar between two mES types. Western blot further revealed that PKCi-derived mES showed lower protein levels of MBD3 (from 1.0 to 0.5) and HDAC2 (from 1.0 to 0.9, *P* < 0.05; Fig. 1B). These changes in RNA and protein expression were maintained (Fig. 1C, D), except for further reduced protein MTA1 (Fig. 1D) at passage 10. When we examined the expression of genes downstream and targets of NuRD [1], we found significantly lower expression of carbonyl reductase 3 (*CBR3*) and high-temperature requirement serine protease A1 (*HTRA1*) mRNA in PKCi-derived mES (Fig. 1E).

### PKCi-derived mES expressed naïve pluripotency genes and possessed germline transmission

Compared with mouse embryonic fibroblasts, PKCi- and 2iL-derived mES showed higher mRNA expression of the pluripotency markers such as *NANOG*, *OCT4*, *C-MYC,* and *SOX2* (Fig. S1A) and naïve-state markers *FGF4*, *NROB1*, *REX1*, and *KLF4* (Fig. S1B) but not primed-state markers *FGF5* and *T* (*P* < 0.05; Fig. S1C).

### Signaling pathways involved in PKCi-derived mES self-renewal

**Fig. 2:**
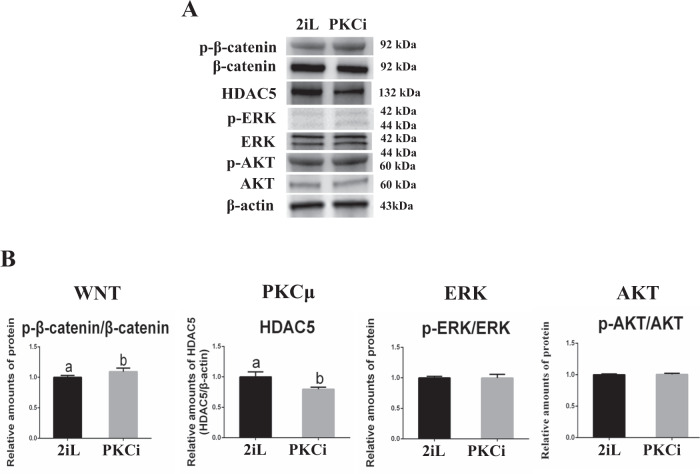
WNT, PKCμ, ERK, and AKT signaling pathways were involved in PKCi-derived mES self-renewal. **A** p-β-catenin/β-catenin, HDAC5, p-ERK/ERK, p-AKT/AKT, and β-actin protein levels were assessed by Western blot in PKCi-derived mES at passage 3, with 2iL-derived mES used as a positive control. **B** Quantitative density analysis showed that PKCi increased protein levels of p-β-catenin/β-catenin and decreased the level of HDAC5. Data were shown as mean ± SD (*n* = 3). The letters a and b indicated significant differences among groups (*P* < 0.05).

As WNT, ERK, and AKT signaling pathways are involved in the self-renewal of 2iL-derived mES [6, 7], we examined key proteins in these pathways in both 2iL- and PKCi-derived mES. Western blot showed that the ratio of phosphorylated (p)-β-catenin/β-catenin was increased in PKCi-derived mES (*P* < 0.01), whereas p-ERK/ERK and p-AKT/AKT ratios were similar between two mES types (Fig. 2A, B). In addition, levels of HDAC5, a key protein in the PKCµ signaling pathway [19], were reduced in PKCi-derived mES (*P* < 0.01; Fig. 2).

### *MBD3* knockdown promoted PKCi-derived mES self-renewal

**Fig. 3:**
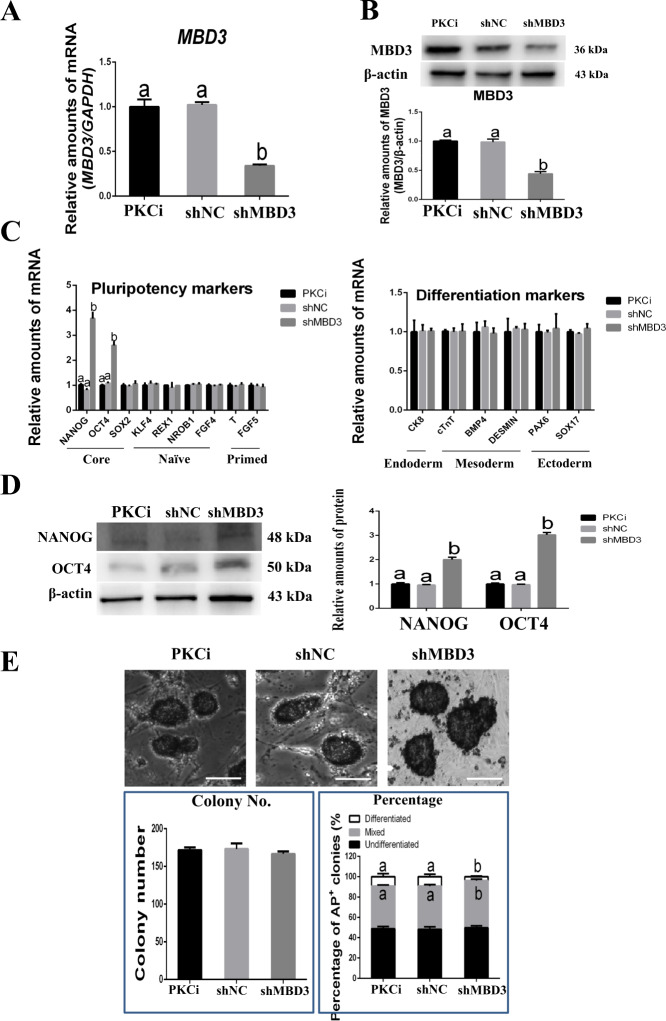
Knockdown of *MBD3* promoted PKCi-derived mES self-renewal. **A** qPCR showed that mRNA levels of *MBD3* decreased after PKCi-derived mES at passage 5 were transfected with shMBD3, with PKCi and shNC groups used as controls. **B** MBD3 and β-actin protein levels were evaluated by Western blot in PKCi-derived mES with *MBD3* knockdown (upper panel). Quantitative density analysis showed that shMBD3 decreased protein levels of MBD3 (lower panel). **C** qPCR showed that compared with the control groups, shMBD3 increased the mRNA levels of pluripotency genes *NANOG* and *OCT4* (left panel) but did not affect the mRNA levels of differentiation genes (right panel). **D** Western blot detection of NANOG, OCT4, and β-actin protein expression in PKCi-derived mES with *MBD3* knockdown (left panel). Quantitative density analysis showed that shMBD3 increased protein levels of NANOG and OCT4 (right panel). **E** Knockdown of *MBD3* did not affect the morphology or AP staining of PKCi-derived mES. Scale bar, 200 μm (upper panel). Knockdown of *MBD3* did not affect the total number of AP-positive colonies (left panel) but increased the percentage of mixed colonies and decreased the percentage of differentiated colonies (right panel). Data were shown as mean ± SD (*n* = 3). The letters a and b indicated significant differences among groups (*P* < 0.05).

To understand the role of MBD3 in mES self-renewal, we knocked down *MBD3* in PKCi-derived mES. Compared with PKCi and shNC control groups, *MBD3* mRNA (from 1.0 to 0.3) (Fig. 3A) and protein (from 1.0 to 0.4) (Fig. 3B) expression were reduced in *MBD3* knocked-down mES (*P* < 0.01). *MBD3* knockdown increased the mRNA levels of pluripotency markers *NANOG* (from 1.0 to 3.7) and *OCT4* (from 1.0 to 2.6) (*P* < 0.01), whereas the expression of other naïve pluripotency, primed-state, and differentiation genes was unchanged (Fig. 3C). As expected, Western blot revealed that the protein levels of NANOG (from 1.0 to 2.0) and OCT4 (from 1.0 to 3.0) were also increased (*P* < 0.01; Fig. 3D). Immunostaining indicated that *MBD3* knockdown did not affect the morphology of mES (Supporting Information Fig. S1D). Although AP staining indicated that compared with PKCi and control groups, *MBD3* knockdown did not affect the total number of AP-positive colonies, the percentage of mixed colonies increased (from 42.1% to 46.4%) and differentiated colonies decreased (from 9.1% to 3.8%), respectively (*P* < 0.05; Fig. 3E).

### Overexpression of *MBD3-*induced mES differentiation

**Fig. 4:**
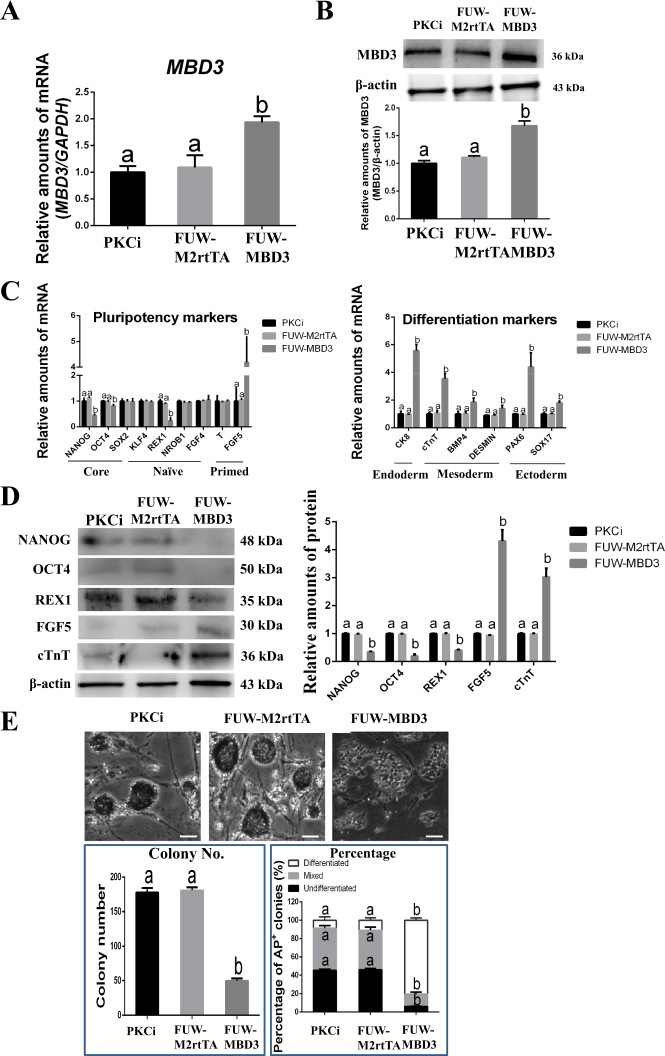
Overexpression of *MBD3*-induced PKCi-derived mES differentiation. **A** qPCR showed that mRNA levels of *MBD3* increased after PKCi-derived mES at passage 5 were transfected with FUW-MBD3, with the PKCi and FUW-M2rtTA plasmid groups used as controls. **B** MBD3 and β-actin protein levels were evaluated by Western blot after PKCi-derived mES were transfected with FUW-MBD3 (upper panel). Quantitative density analysis showed that protein levels of MBD3 increased after transfection with FUW-MBD3 (lower panel). **C** qPCR showed decreased mRNA levels of pluripotency genes *NANOG* and *OCT4* and naïve-state marker *REX1*, but increased mRNA levels of primed-state marker *FGF5* after *MBD3* was overexpressed (left panel). *MBD3* overexpression also increased mRNA levels of the endoderm marker *CK8*, mesoderm markers *cTnT*, *BMP4*, and *DESMIN*, and ectoderm markers *PAX6* and *SOX17* (right panel). **D** Western blot detection of NANOG, OCT4, REX1, FGF5, cTnT, and β-actin protein expression after *MBD3* overexpression (left panel). Quantitative density analysis showed that *MBD3* overexpression decreased protein levels of NANOG, OCT4, and REX1, but increased protein levels of FGF5 and cTnT (right panel). **E** *MBD3* overexpression induced PKCi-derived mES differentiation and resulted in a loss of AP staining. Scale bar, 200 μm (upper panel). Overexpression of *MBD3* reduced the total number of AP-positive colonies (left panel), decreased the percentage of undifferentiated and mixed colonies, and increased the percentage of differentiated colonies (right panel). Data were shown as mean ± SD (*n* = 3). The letters a and b indicated significant differences among groups (*P* < 0.05).

Compared with PKCi and FUW-M2rtTA control groups, *MBD3* overexpression increased mRNA (from 1.0 to 2.0; Fig. 4A) and protein (from 1.0 to 1.4, *P* < 0.05; Fig. 4B) levels of MBD3 in PKCi-derived mES. qPCR showed that *MBD3* overexpression significantly decreased mRNA levels of the pluripotency markers *NANOG* (from 1.0 to 0.5) and *OCT4* (from 1.0 to 0.8) and the naïve-state marker *REX1* (from 1.0 to 0.2) and increased mRNAs levels of the primed-state marker *FGF5* (from 1.0 to 4.2). mRNA levels of the differentiation markers *CK8* (from 1.0 to 5.6), *cTnT* (from 1.0 to 3.6), *BMP4* (from 1.0 to 1.9), *DESMIN* (from 1.0 to 1.4), *PAX6* (from 1.0 to 4.4), and *SOX17* (from 1.0 to 1.8) were increased in *MBD3*-overexpressing mES (*P* < 0.05; Fig. 4C). Also, *MBD3* overexpression decreased protein levels of NANOG (from 1.0 to 0.3) and OCT4 (from 1.0 to 0.2) and increased protein levels of cTnT (from 1.0 to 3.0, *P* < 0.05; Fig. 4D).

*MBD3* overexpression induced mES differentiation into flat cells that did not express NANOG (Supporting Information Fig. S2C). Compared with the PKCi control group, *MBD3* overexpression reduced the total number of AP-positive colonies (from 178 to 50) and the percentage of undifferentiated (from 45.4% to 6.1%) and mixed (from 46.0% to 13.4%) colonies, but increased the percentage of differentiated colonies (from 8.7% to 80.5%, *P* < 0.05; Fig. 4E).

### Removal of PKC inhibitor increased *MBD3* expression and mES differentiation, which was partially rescued by *MBD3* knockdown

**Fig. 5:**
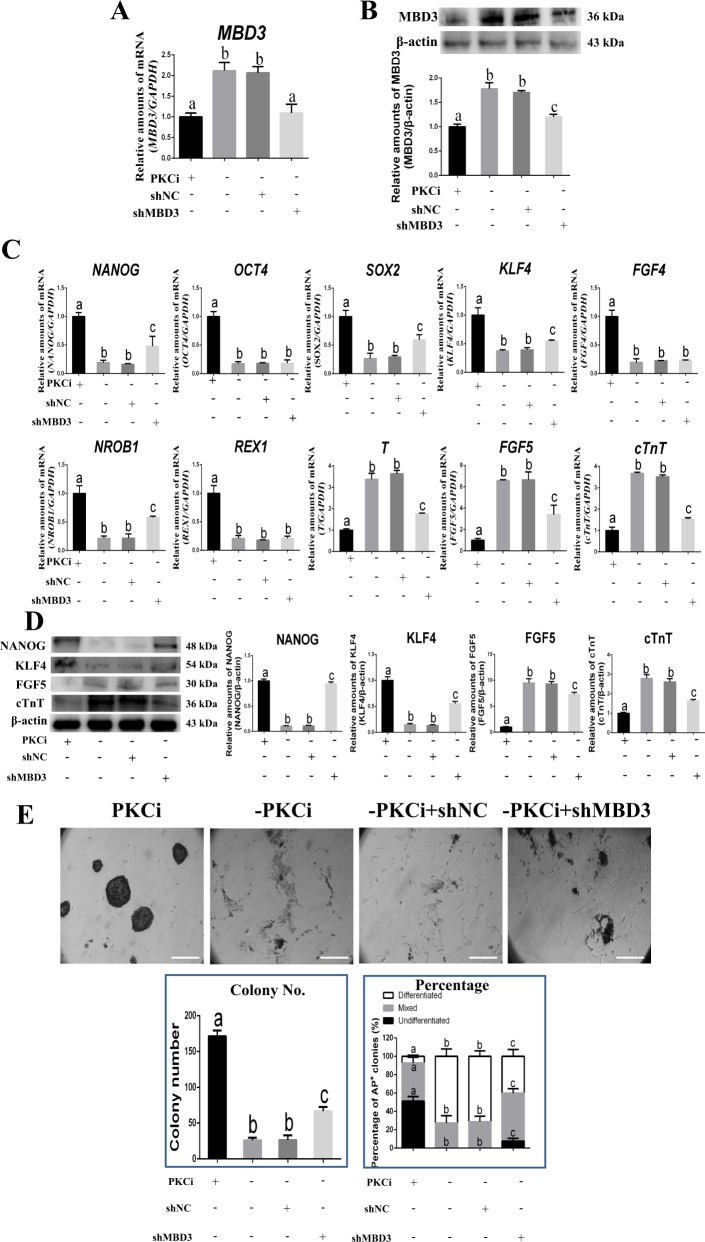
*MBD3* knockdown partially reversed the upregulation of *MBD3* and cell differentiation induced by the removal of PKC inhibitor. **A** *MBD3* RNA expression increased in mES at passage 5 when the PKC inhibitor was removed for 48 h and was partially reversed by *MBD3* knockdown, with the PKCi and PKCi removal plus shNC transfection groups used as controls. **B** MBD3 and β-actin protein levels were evaluated by Western blot (upper panel). Quantitative density analysis showed that MBD3 protein levels increased after PKC inhibitor was removed, which was partially reversed by *MBD3* knockdown (lower panel). **C** PKC inhibitor removal decreased mRNA levels of pluripotency genes *NANOG*, *OCT4*, and *SOX2* and naïve-state markers *KLF4, FGF4, REX1,* and *NROB1*, whereas *MBD3* knockdown partially increased the mRNA levels of pluripotency gene, *NANOG, SOX2, KLF4,* and *NROB1*. PKC inhibitor removal increased mRNA levels of primed-state markers *T* and *FGF5* and the mesoderm gene *cTnT*, whereas *MBD3* knockdown partially decreased mRNA levels of *T*, *FGF5*, and *cTnT*. **D** NANOG, KLF4, FGF5, cTnT, and β-actin levels were detected by Western blot (left panel). Quantitative density analysis showed that NANOG and KLF4 protein levels decreased and FGF5 and cTnT protein levels increased after PKC inhibitor removal, whereas NANOG and KLF4 protein levels partially increased, but FGF5 and cTnT levels partially decreased after *MBD3* knockdown (right panel). **E** After PKC inhibitor removal, mES differentiated and did not show AP staining, whereas *MBD3* knockdown partially recovered mES morphology and restored AP staining. Scale bar, 200 μm (upper panel). PKC inhibitor removal decreased the total number of AP-positive colonies, which was partially reversed by *MBD3* knockdown (left panel). *MBD3* knockdown partially reversed the decrease in the percentage of mixed and undifferentiated colonies and the increase in the percentage of differentiated colonies induced by PKC inhibitor removal (right panel). Data were shown as mean ± SD (*n* = 3). The letters a, b, and c indicated significant differences among groups (*P* < 0.05).

When the PKC inhibitor Gӧ6983 was removed from the PKCi culture medium for 48 h, *MBD3* mRNA (from 1.0 to 2.1) and protein (from 1.0 to 1.7) levels increased in mES (*P* < 0.05; Fig. 5A, B). This change was associated with decreased mRNA levels of pluripotency markers *NANOG* (from 1.0 to 0.2), *OCT4* (from 1.0 to 0.2), and *SOX2* (from 1.0 to 0.3), decreased levels of naïve-state markers *KLF4* (from 1.0 to 0.4), *FGF4* (from 1.0 to 0.2), *NROB1* (from 1.0 to 0.2), and *REX1* (from 1.0 to 0.2); and increased mRNA levels of primed-state markers *T* (from 1.0 to 3.4) and *FGF5* (from 1.0 to 6.6) and differentiation genes *cTnT* (from 1.0 to 3.7, *P* < 0.05; Fig. 5C), *CK8* (from 1.0 to 2.4), *BMP4* (from 1.0 to 3.6), *DESMIN* (from 1.0 to 1.9), *PAX6* (from 1.0 to 6.0), and *SOX17* (from 1.0 to 4.4, *P* < 0.05; Supporting Information Fig. S3E). By contrast, compared with PKCi-derived control mES (normalized as 1.0), *MBD3* knockdown in mES cultured without PKC inhibitor showed reduced *MBD3* mRNA (from 2.1 to 1.1) and protein (from 1.7 to 1.3) levels, albeit its protein still higher than those in PKCi-derived mES (*P* < 0.05, Fig. 5B). In addition, *MBD3* knockdown increased mRNA levels of pluripotent markers *NANOG* (from 0.2 to 0.5) and *SOX2* (from 0.3 to 0.6) and naïve-state markers *KLF4* (from 0.4 to 0.5) and *NROB1* (from 0.2 to 0.6, *P* < 0.05); decreased mRNA levels of primed-state markers *T* (from 3.4 to 1.8) and *FGF5* (from 6.6 to 3.4) and differentiation genes *cTnT* (from 3.7 to 1.5, Fig. 5C), *SOX17* (from 4.4 to 2.9), and *BMP4* (from 3.6 to 2.2); and recovered mRNA levels of *PAX6* to initial PKCi levels (from 6.0 to 2.8; Supporting Information Fig. S3E).

Western blot showed that the removal of PKC inhibitor in culture significantly reduced mES protein levels of NANOG (from 1.0 to 0.2) and KLF4 (from 1.0 to 0.4) and increased protein levels of FGF5 (from 1.0 to 6.2) and cTnT (from 1.0 to 3.4) (Fig. 5C). However, *MBD3* knockdown in mES cultured without PKC inhibitor partially increased protein levels of NANOG (from 0.2 to 0.4) and KLF4 (from 0.4 to 0.6) and decreased protein levels of FGF5 (from 6.2 to 3.0) and cTnT (from 3.4 to 1.6), but there was not full recovery to initial PKCi levels (*P* < 0.05; Fig. 5D). Immunostaining showed that after PKC inhibitor removal, mES differentiated into flattened cells that did not express NANOG, whereas knockdown of *MBD3* partially prevented mES differentiation and induced weak NANOG expression compared with shNCs cultured without PKC inhibitor (*P* < 0.05; Supporting Information Fig. S3A–S3D).

PKC inhibitor removal induced mES differentiation, and a few cell colonies existed that were negative for AP staining (Fig. 5E). Quantitative analysis showed that PKC inhibitor removal significantly reduced the number of AP-positive colonies (from 171 to 26), and the percentage of mixed (from 42.5% to 27.4%) and undifferentiated (from 51.3% to 0%) colonies and increased the percentage of differentiated colonies (from 7.2% to 72.6%; Fig. 5E). However, *MBD3* knockdown partially recovered mES colony morphology, increased the number of AP-positive colonies (from 26 to 67) and percentage of undifferentiated (from 0% to 7.5%) and mixed (from 27.4% to 52.4%) colonies, and decreased the percentage of differentiated colonies (from 72.6% to 40%, *P* < 0.05; Fig. 5E).

## Discussion

We demonstrated that inhibition of the PKC-signaling pathway by Gӧ6983 reduced the expression of NuRD components at both RNA (*MBD3*, *HDAC1/ HDAC2*, *MTA1, RbAP46/RbAP48*, and *p66α*) and protein (MBD3 and HDAC2 at passage 3, further MTA1 at passage 10) levels in mES. The PKC-signaling pathway is a complex signal transduction network that participates in other signaling pathways, such as MEK/ERK [20], CREB [21], PKCζ-NF-ĸB [8, 9], PKCµ [19], and GSK3β [22]. Small molecules, such as PD0325901 and CHIR99021 inhibit MEK/ERK and GSK3β pathways, which together with activation of JAK-STAT3 maintain mES naïve pluripotency [5, 7]. In our study, we found comparable ratios of p-ERK/ERK and p-AKT/AKT between 2iL- and PKCi-derived mES, suggesting that inhibition of PKC by the small molecule Gӧ6983 plays a similar role as PD0325901 in suppressing MEK/ERK pathway. On the other hand, we observed an elevated ratio of p-β-catenin/β-catenin in PKCi-derived mES, implying that Gӧ6983 inhibits GSK3-β more effectively than CHIR99021. A previous study [8] reported that GSK3, ERK1/2, AKT, and their respective downstream target genes β-catenin, RSK1, and STAT3 were not phosphorylated in the PKCi system, suggesting that PKCi regulates mES self-renewal independently of traditional signaling pathways. However, we found that the p-ERK/ERK ratio was lower in the PKCi system compared with that after PKC inhibitor removal, indicating that the ERK-signaling pathway is involved in PKCi-derived mES self-renewal. Interestingly, in human ES [23], FGF2 activates PI3K/AKT, MEK/ERK1/2, and PKC isoforms (i.e., PKCδ/ε/ζ), resulting in phosphorylation of GSK-3β, and that activation of AKT signaling promotes self-renewal whereas activation of GSK3β and ERK1/2 induces differentiation. In addition, PKCi system is involved in the downregulation of HDAC5, a key protein in the PKCµ pathway, which maintains mES self-renewal and prevents cell lineage commitment and differentiation, moreover, the inhibition of PKCµ suppresses myoblast differentiation by inhibiting MYOD and myocyte-specific enhancer factor 2C [19]. Key transcription factors are pivotal for maintaining gene expression patterns, chromatin structure, and the epigenetic landscape allowing ES self-renewal [5, 7, 8]. These transcription factors affect the expression of other repressive regulatory factors, including the NuRD complex. Although transcription levels of *HDAC1*, *MTA1*, *CHD3*, *RbAP46*, and *RbAP48* were changed, protein levels were not affected, probably due to the regulation of translation and the lower threshold of RNA required for translation. The mechanism that PKCi down-regulates the expression of *MBD3* and other genes is unknown. In the present study, a reduction in NuRD expression was confirmed by decreased expression of NuRD targeted *CBR3* and *HTRA1* mRNA [1] in PKCi-derived mES. It is assumed that NuRD targeted gene may play a role in regulating the naïve pluripotency.

Previous studies using Gӧ6983, a selective inhibitor of PKC isoforms, show that the PKCζ–NF-ĸB–miRNA-21/miRNA-29 regulatory signaling axis plays a critical role in inducing mES lineage commitment and is capable of maintaining mES and rat ES-specific epigenetic modifications and self-renewal [8, 9]. Our study confirms this finding by showing that knockdown of PKCζ partially rescued the amount of AP-positive mES colonies upon the removal of PKC inhibitor (Fig. S2A, S2B), indicating that inhibition of the NF-ĸB signaling pathway plays an important role in maintaining PKCi-derived mES pluripotency.

The NuRD complex is an abundant and conserved remodeling complex repressor that increases nucleosome density and fine-tunes differential gene expression, even at active transcription sites [1]. MBD3, a key NuRD subunit, is necessary for the development of pluripotent cells, as MBD3-depleted mES are viable but fail to form stable NuRD complexes, which severely affect their cell commitment and differentiation capacities [12]. In the present study, we found that MBD3 plays an important role in mES self-renewal and differentiation. In the PKCi system, knockdown of *MBD3* increased the expression of the pluripotency genes *NANOG* and *OCT4*, thereby promoting mES self-renewal. Interestingly, knockdown of *MBD3* did not affect other pluripotency genes such as *KLF4*, *SOX2*, *REX1*, *NROB1*, or *FGF4*. In particular, a previous study reported that KLF4, a naïve-state marker and direct downstream target of LIF/STAT3 [24, 25], mediates the self-renewal-promoting effects of GBX2 in ESs [26]. Therefore, we assume that the LIF/STAT3-signaling pathway does not play a dominant role in the PKCi system. In fact, a previous study found mES devoid of STAT3, suggesting that LIF/STAT3 signaling does not instruct ES self-renewal but may instead act in unrefined culture conditions [7]. By contrast, we observed that MBD3 overexpression and/or PKC inhibitor removal increased MBD3 mRNA and protein levels, downregulated mRNA levels of pluripotency genes such as *NANOG* and *OCT4* and naïve-state genes such as *REX1*, and upregulated the expression of differentiation genes such as the endoderm marker *CK8*, mesoderm markers *cTnT*, *BMP4*, and *DESMIN*, and ectoderm markers *PAX6* and *SOX17*. Overexpression of MBD3 also reduced protein levels of NANOG, OCT4, and REX1 but increased protein levels of the primed-state marker FGF5 and differentiation marker cTnT. As a result, the number of ES colonies decreased due to cell differentiation. Furthermore, *MBD3* knockdown by RNA interference partially reversed the cell differentiation induced by PKC inhibitor removal. Therefore, our findings support previous findings that the repression of genes related to lineage commitment and cell differentiation is necessary for maintaining mES self-renewal [7, 27]. MBD3 directly regulates pluripotency gene transcription in ESs [16], with one study reporting that MBD3 and Brg1 antagonistically regulate a common set of genes by regulating promoter nucleosome occupancy to maintain pluripotency of embryonic stem cells in mouse [28]. The balance between repressive NuRD and activating chromatin remodeling complex BAF may finely tune gene expression specific to stem cell pluripotency [1]. As a result, mES exhibit a transient cell state poised for cell lineage differentiation during development [5, 29].

In summary, we found that the PKCi culture system reduced the expression of the repressive and epigenetic regulatory NuRD complex and its MBD3, HDAC2, and MTA1 subunits in mES. This reduction in NuRD promoted the expression of pluripotency genes that maintain the naïve state of mES. Functional knockdown and overexpression experiments revealed that MBD3, a key NuRD subunit, plays an important role in regulating the expression of genes responsible for mES pluripotency. This demonstration of the transcriptional/translational regulatory effects of the NuRD complex on a wide spectrum of developmental genes increases our understanding of ES pluripotency, lineage commitment, and differentiation.

## Materials and methods

### Chemicals and reagents

Unless otherwise stated, all chemicals and reagents were purchased from Sigma-Aldrich (St. Louis, MO, USA).

### Animal maintenance, hormone-induced superovulation, and blastocyst collection

Animal experimental protocols were approved by the Animal Care and Use Committee of Nanjing Normal University (NSD-2013-30) and were performed according to guidelines from the US National Institutes of Health. At 6–8 weeks of age, female C57BL/6J mice were intraperitoneally injected with 7.5 IU pregnant mare serum gonadotropin (Ningbo Second Hormone Factory, China). Forty-eight hours later, mice were intraperitoneally injected with 7.5 IU human chorionic gonadotropin (Ningbo Second Hormone Factory) and mated with males (1:1). Blastocysts were flushed from the uterus with M2 medium on day 3.5.

### De novo derivation of mES

Blastocysts were seeded on Mitomycin C-treated mouse embryonic fibroblasts on 0.1% gelatin-coated plates (ES-006-B, Millipore, USA) with PKC inhibitor (5 μM Gӧ6983, 133053-19-7, Selleck, USA) in basic culture medium supplemented with Dulbecco’s modified Eagle’s medium (DMEM; 10829018, Gibco, USA) containing 15% knockout serum replacement (10828028, Gibco), 1% penicillin/streptomycin (SV30010, HyClone, USA), 2 mM glutamine (35050061, Gibco), 1 mM sodium pyruvate (11360088, Gibco), and 0.1 mM 2-mercaptoethanol (ES-007-E, Millipore). After culture for 7 days, outgrowths were collected and digested into single-cell suspensions with accutase (A1110501, Gibco) and re-seeded in new plates coated with feeder cells. mES passaging was performed by incubating colonies with accutase, followed by plating at a density of 1 × 10^3^ cells/cm^2^ into a new 24-well plate coated with new feeder cells at 3- to 4-day intervals. Collected mES were frozen in a cryopreservation medium with 90% fetal bovine serum (FBS; SH30070.03, HyClone) and 10% dimethyl sulfoxide (D5879) and stored in liquid nitrogen.

### *MBD3* overexpression in mES

FUW-MBD3 (#52356) and control FUW-M2rtTA (#20342) were purchased from Addgene. Lentiviral infection and overexpression were performed as previously described [30]. Briefly, 293T cells were cultured for 2 days in DMEM (C11995500BT, Gibco) supplemented with 10% FBS (v/v) at 37 °C in 5% CO_2_. At 70–80% confluency, cells were transfected with FUW-MBD3 or control FUW-M2rtTA along with the viral packaging plasmids psPAX and pMD2.G (5:3:2) with Lipofectamine 2000 reagent (1947415, Invitrogen, USA) at a 1:2 ratio of DNA (g) to Lipofectamine 2000 (μL). After 6 h, the medium was changed. After transfection for 48 h, viral supernatants were collected and filtered (0.45-µm pore size; Millipore) to infect mES. For lentiviral transfection, mES at 70–80% confluency were infected with filtered viral supernatants (FUW-MBD3 or FUW-M2rtTA) supplemented with an equal volume of fresh PKCi medium. Twelve hours later, mES were repeatedly infected with viral supernatants up to four times within 48 h as needed.

### RNA interference in mES

Lentiviral supernatants containing short hairpin RNA (shRNA) targeting mouse *MBD3* mRNA (shMBD3), PKCζ KD1, or PKCζ KD2 or an shRNA negative control (shNC) were purchased from GenePharma (Shanghai, China). The shPKCζ KD1 target sequence was GGGACGAAGTGCTCATCATTC, shPKCζKD2 was GGATCGACCAGTCCGAATTTG, shMBD3 was GCCTCCTTATCATAGGACAAG, and shNC was TTCTCCGAACGTGTCACGT. RNA interference was performed according to the manufacturer’s instructions. Briefly, mES were cultured for 1–2 days with PKCi medium, after which lentiviral diluent was added to the culture medium and incubated overnight at 37 °C in 5% CO_2_. After 24 h of incubation, the medium was changed to fresh PKCi medium, and incubation continued for another 24 h at 37 °C in 5% CO_2_. Infected mES were cultured in PKCi-free ES medium for 48 h and collected for further analysis and Western blot assay. shNC infection was used as a knock-down control (−PKCi + shNC).

### qPCR

Total RNA was extracted from mES with Trizol reagent (T9424). Reverse transcription reactions were performed with 1 μg RNA using HiScript II Reverse Transcriptase (R223-01, Vazyme, China). Complementary DNA was used as a template, and 2xSYBR Green Fast qPCR Mix with High Rox (RM21206, ABclonal, China) was used for the qPCR reaction. The qPCR primers are shown in Table 1. Individual gene expression was normalized to *GAPDH* expression, and values from the PKCi group were defined as 1.0 for all gene expression levels.

**Table 1:** Sequences of primers used for qRT-PCR.

Gene symbol	Forward primer (5’–3’)	Reverse primer (5’–3’)	PCR condition	Size (bp)
*NANOG*	AGAAGTACCTCAGCCTCCAGC	AGATGCGTTCACCAGATAGCC	95 °C 15 s, annealing/extension 60 °C 60 s, 40 cycles	224
*OCT4*	GAGGAAGCCGACAACAATGAG	TGTGAGTGATCTGCTGTAGGGAG	95 °C 15 s, annealing/extension 60 °C 60 s, 40 cycles	163
*C-MYC*	GAGCCCACCACACACATTCT	GGGGAACAGATTCTGGCAGT	95 °C 1 s, annealing/extension 60 °C 60 s, 40 cycles	185
*SOX2*	GGTTACCTCTTCCTCCCACTCCAGG	TGTGCCGTTAATGGCCGTGCC	95 °C 1 s, annealing/extension 60 °C 60 s, 40 cycles	172
*FGF4*	GTGGTGAGCATCTTCGGAGTGG	GCGTAGGATTCGTAGGCGTTGT	95 °C 15 s, annealing/extension 60 °C 60 s, 40 cycles	146
*NROB1*	TTGACACCAAAGAGTATGCCTATC	AAGGGCACTGTTCAGTTCAGC	95 °C 15 s, annealing/extension 60 °C 60 s, 40 cycles	182
*REX1*	GCATCGCCCACAGCCCATCACT	CTGGTTGGACGAACAGAACTTGA	95 °C 15 s, annealing/extension 60 °C 60 s, 40 cycles	143
*KLF4*	ACTGTCACCCTGGCCTGCCTCT	CCCTCTTTGGCTTGGGCTCCT	95 °C 15 s, annealing/extension 60 °C 60 s, 40 cycles	165
*FGF5*	GCTCGGAACATAGCAGTTTC	CCGTAAATTTGGCTTAACACAC	95 °C 15 s, annealing/extension 60 °C 60 s, 40 cycles	151
*T*	ACCTATGCGGACAATTCATC	CAGACCAGAGACTGGGATAC	95 °C 15 s, annealing/extension 60 °C 60 s, 40 cycles	155
*CK8*	AGATTGAAGCCCTCAAAGG	AGCTTGACGTTCATAAGCTC	95 °C 15 s, annealing/extension 60 °C 60 s, 40 cycles	187
*BMP4*	ATCACGAAGAACATCTGGAG	GAGATCACCTCATTCTCTGG	95 °C 15 s, annealing/extension 60 °C 60 s, 40 cycles	100
*DESMIN*	AGAAAGTGCATGAAGAGGAG	CCTCAGAGATGTTCTTAGCC	95 °C 15 s, annealing/extension 60 °C 60 s, 40 cycles	156
*PAX6*	CGGAAGCTGCAAAGAAATAG	CCTGTATTCTTGCTTCAGGT	95 °C 15 s, annealing/extension 60 °C 60 s, 40 cycles	145
*SOX17*	GCGTGGAGCAGGACCCGGCTTTCTT	GGACACTGCATAGTCCGAGACTGG	95 °C 15 s, annealing/extension 60 °C 60 s, 40 cycles	101
*cTnT*	AGACTGGAGTGAAGAAGAGGAGGAC	CTGGGCTTGGGTTTGGTGT	95 °C 15 s, annealing/extension 60 °C 60 s, 40 cycles	186
*HDAC1*	ACAAAGCCAATGCTGAGGAGA	TCAAACAAGCCATCAAACACC	95 °C 15 s, annealing/extension 60 °C 60 s, 40 cycles	154
*HDAC2*	CGGTGTTTGATGGACTCTTTG	AGAACCCTGATGCTTCTGACT	95 °C 15 s, annealing/extension 60 °C 60 s, 40 cycles	150
*MBD3*	CAGCCATTGCGAGTGCTCTAC	CTGTCACCATGAAGGCTTTGC	95 °C 15 s, annealing/extension 60 °C 60 s, 40 cycles	129
*MTA1*	ACAAGACAGCCAACGGGAATG	CAACTGCCGAGACAGGAACAG	95 °C 15 s, annealing/extension 60 °C 60 s, 40 cycles	200
*CHD3*	AGAGTGGAGGCAGCGAGTATGG	TCAGAAGCAAGGTTGCGGATG	95 °C 15 s, annealing/extension 60 °C 60 s, 40 cycles	156
*RbAP46*	TGCTGCATGAGTCCTTGTTTG	CAGAATGAACTCGCTGTAGGG	95 °C 15 s, annealing/extension 60 °C 60 s, 40 cycles	158
*RbAP48*	CATACAGCAGTAGTGGAGGACG	TGTGAGCATCAACCGAGTGGC	95 °C 15 s, annealing/extension 60 °C 60 s, 40 cycles	186
*DOC1*	GGAAGTGTCCACTCACCCTCTACT	ACTCTGAGGCACCTGGCTATTT	95 °C 15 s, annealing/extension 60 °C 60 s, 40 cycles	126
*P66α*	TGGCAAGACCTCACTTCAGACC	GACATTGGCGACACGGATAA	95 °C 15 s, annealing/extension 60 °C 60 s, 40 cycles	284
*P66β*	CTCGCCTGGTGCTGCTAAAGA	ATGGCTGAACAATAGATGCTG	95 °C 15 s, annealing/extension 60 °C 60 s, 40 cycles	105
*GAPDH*	GTGGCAAAGTGGAGATTGTTG	CTCCTGGAAGATGGTGATGG	95 °C 15 s, annealing/extension 60 °C 60 s, 40 cycles	164

### Western blot

mES total protein was collected with lysis buffer and quantified using a BCA Kit (GK5012, Beyotime Biotechnology, China). Western blot analysis was performed as previously described [31]. Briefly, 6 μg total protein was loaded into each well of a 12% gel for SDS–PAGE. After electrophoresis, separated proteins were transferred to a polyvinylidene fluoride membrane (03010040001, Roche, Basel, Switzerland) by electrotransfer. Membranes were blocked with 5% non-fat powdered milk (A600669, Sangon Biotech, China) for 1 h, after which membranes were washed with tris-buffered saline containingTween 20 (TBST; 9005-64-5, Sangon Biotech) and incubated with anti-p-β-catenin (1:1000, 9561T, Cell Signaling Technology, CST, USA), anti-β-catenin (1:1000, A19657, ABclonal, China), anti-p-ERK (1:1000, 3510, CST), anti-ERK (1:1000, 4695, CST), anti-p-AKT (1:1000, AP1214, ABclonal), anti-AKT (1:1000, A17909, ABclonal), anti-HDAC5 (A0632, ABclonal), anti-HDAC1 (1:1000, A0238, ABclonal), anti-HDAC2 (1:1000, A2084, ABclonal), anti-MTA1 (1:1000, A16085, ABclonal), anti-MBD3 (1:1000, A2251, Lot 0046180101, ABclonal), anti-CHD3 (1:1000, A2221, ABclonal), anti-RbAP46 (1:1000, A6967, ABclonal), anti-RbAP48 (1:1000, A13934, ABclonal), anti-NANOG (1:1000, Lannuo Biotechnology, China), anti-OCT4 (1:1000, Lannuo Biotechnology), anti-REX1 (1:1000, Lannuo Biotechnology), anti-FGF5 (1:1000, ab88118, Abcam), or anti-cTnT (1:1000, A4914, ABclonal) antibodies overnight at 4 °C. The next day, membranes were washed with TBST and incubated with horseradish peroxidase-conjugated goat anti-rabbit secondary antibody (1:5000, BS13278, Bioworld) for 1 h. After washing, membranes were processed using an enhanced chemiluminescence reagent (E411-04, Vazyme), and protein bands were visualized using a LAS-4000 imager (Tanon, Shanghai, China). β-actin (1:1000, AC026, ABclonal) was used as an internal control. Target protein expression levels were normalized to those of β-actin. Values from the PKCi group were defined as 1.0 and were used for comparisons with other treatments.

### Immunocytochemical staining for pluripotency markers

Immunostaining experiments were performed with the pluripotency marker NANOG (1:200, Lannuo Biotechnologies). Briefly, mES were washed with Dulbecco’s phosphate-buffered saline (DPBS) three times, fixed with 4% paraformaldehyde for 10 min at room temperature, washed with DPBS three times, incubated with 0.2% Triton-X 100 (Solarbio, China) for 5 min, washed with DPBS three times, incubated with 2% FBS (HyClone) for 30 min at room temperature to block nonspecific binding, and incubated with primary antibodies at 4 °C overnight. The next day, cells were washed with DPBS three times, incubated with secondary antibody conjugated with FITC (goat anti-rabbit IgG (H + L), 1:300, AS011, ABclonal) for 2 h at room temperature, washed with DPBS, and stained with 100 ng/ml DAPI (SunShine, China) for 10 min at room temperature in the dark. Finally, after washing with DPBS, cells were observed under an inverted fluorescence microscope.

### Alkaline phosphatase staining

mES were washed with DPBS three times, fixed with 4% paraformaldehyde for 10 min at room temperature, washed with DPBS three times, incubated with nitro blue tetrazolium and 5-bromo-4-chloro-3-indolylphosphate substrates (REF 11745832910, Roche, Switzerland) for 20–30 min at room temperature, washed with DPBS, and observed under an inverted microscope.

### Statistical analysis

All experiments were repeated at least three times. mES derived from either PKCi or LIF-2i were from different blastocysts, but mES after derivation in two conditions were the same strains to exclude the possibility of variations. Data were analyzed using SPSS version 18.0 (SPSS, Inc., Chicago, IL, USA) and were shown as mean ± standard deviation (SD). Statistical comparisons were performed using analysis of variance (ANOVA). The letters a, b, and c indicated significant differences among groups (*P* < 0.05).

## Supplementary information

Supplementary-final

Supplementary figures

Original Data File

## Data Availability

All data related to this paper may be requested from the corresponding author.
