# Supplementary material for: Nucleosome remodeling and deacetylation complex and MBD3 influence mouse embryonic stem cell naïve pluripotency under inhibition of protein kinase C
Source: Cell Death Discov. 2022 Aug 1;8:344. doi: 10.1038/s41420-022-01131-0 (PMC9343426; doi:10.1038/s41420-022-01131-0)
Supplement: Supplementary file 1 — Supplementary-final [file 41420_2022_1131_MOESM1_ESM.docx]

**Nucleosome Remodeling and Deacetylation Complex and MBD3 Influence Mouse Naïve Embryonic Stem Cell Pluripotency under Inhibition of Protein Kinase C**

**Supplementary Data**

**Figure S1. PKCi-derived mES expressed pluripotency and naïve-state markers but not primed-state markers.** (**A**) qPCR showed that compared with the mouse embryonic fibroblast (MEF) control group, mRNA levels of pluripotency markers *NANOG*, *OCT4*, *C-MYC,* and *SOX2* were highly expressed in PKCi-derived mES at passage 3, with 2iL-derived mES used as a positive control. (**B**) qPCR showed that compared with MEF control, mRNA levels of naïve-state markers *FGF4, NROB1, REX1,* and *KLF4* were highly expressed. (**C**) qPCR showed that compared with the MEF group, mRNA levels of primed-state markers *FGF5* and *T* were minimally expressed. (**D**) Immunostaining showed that shMBD3 did not affect the morphology of PKCi-derived mES at passage 5, which expressed NANOG, with PKCi and shNC groups used as controls. Scale bar, 200 μm. Data were shown as mean ± SD (n=3). The letters a and b indicated significant differences among groups (*P*<0.05).

**Figure S2. Knockdown of PKCζ was sufficient to maintain mES self-renewal.** (**A**) PKC inhibitor removal for 48 h induced passage 5 mES differentiation, which was reversed by knockdown of PKCζ with KD1 or KD2, with PKCi used as a positive control (upper panel). Few mES showed AP staining after PKCi removal. Scale bar, 200 μm. (**B**) Compared with PKCi, PKCi removal decreased the percentage of AP-positive colonies, which was partially reversed by knockdown of PKCζ with KD1 or KD2. (**C**) Immunostaining showed that MBD3 overexpression induced PKCi-derived differentiation of passage 5 mES, which did not express NANOG. Scale bar, 200 μm. Data were shown as mean ± SD (n=3). The letters a, b, c, and d indicated significant differences among groups (*P*<0.05).

**Figure S3. MBD3 knockdown partially reversed the upregulation of differentiation genes induced by removal of PKC inhibitor.** (**A–D**) Immunostaining showed that PKC inhibitor removal for 48 h induced differentiation of passage 5 mES, which did not express NANOG. MBD3 knockdown partially reversed this differentiation and loss of NANOG expression, with PKCi used as a positive control and shNC, with PKCi removal used as a knockdown control. Scale bar, 200 μm. (**E**) qPCR showed that compared with PKCi, removal of PKCi increased mRNA levels of differentiation genes *CK8*, *BMP4*, *DESMIN*, *PAX6,* and *SOX17*, whereas MBD3 knockdown partially decreased levels of *BMP4*, *PAX6,* and *SOX17*. Data were shown as mean ± SD (n=3). The letters a, b, and c indicated significant differences among groups (*P*<0.05).
