# Supplementary material for: Nucleosome remodeling and deacetylation complex and MBD3 influence mouse embryonic stem cell naïve pluripotency under inhibition of protein kinase C
Source: Cell Death Discov. 2022 Aug 1;8:344. doi: 10.1038/s41420-022-01131-0 (PMC9343426; doi:10.1038/s41420-022-01131-0)
Supplement: Supplementary file 2 — Supplementary figures [file 41420_2022_1131_MOESM2_ESM.pptx]

## Slide 1
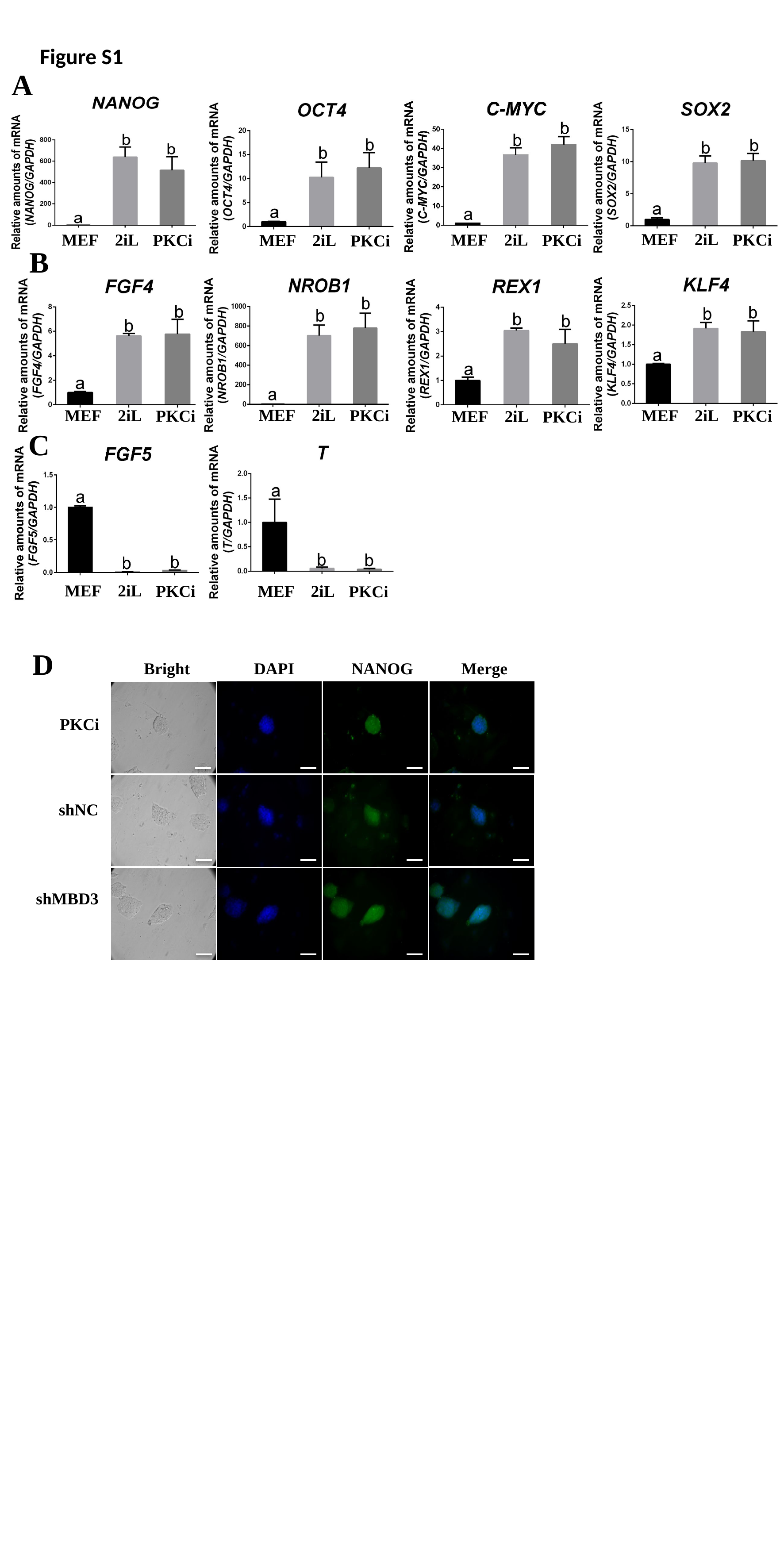

Figure S1
A
MEF
2iL
MEF
2iL
MEF
2iL
PKCi
MEF
2iL
PKCi
PKCi
PKCi
B
MEF
2iL
MEF
2iL
PKCi
MEF
2iL
PKCi
PKCi
MEF
2iL
PKCi
C
MEF
2iL
MEF
2iL
PKCi
PKCi
D
Bright
DAPI
NANOG
Merge
PKCi
shNC
shMBD3

## Slide 2
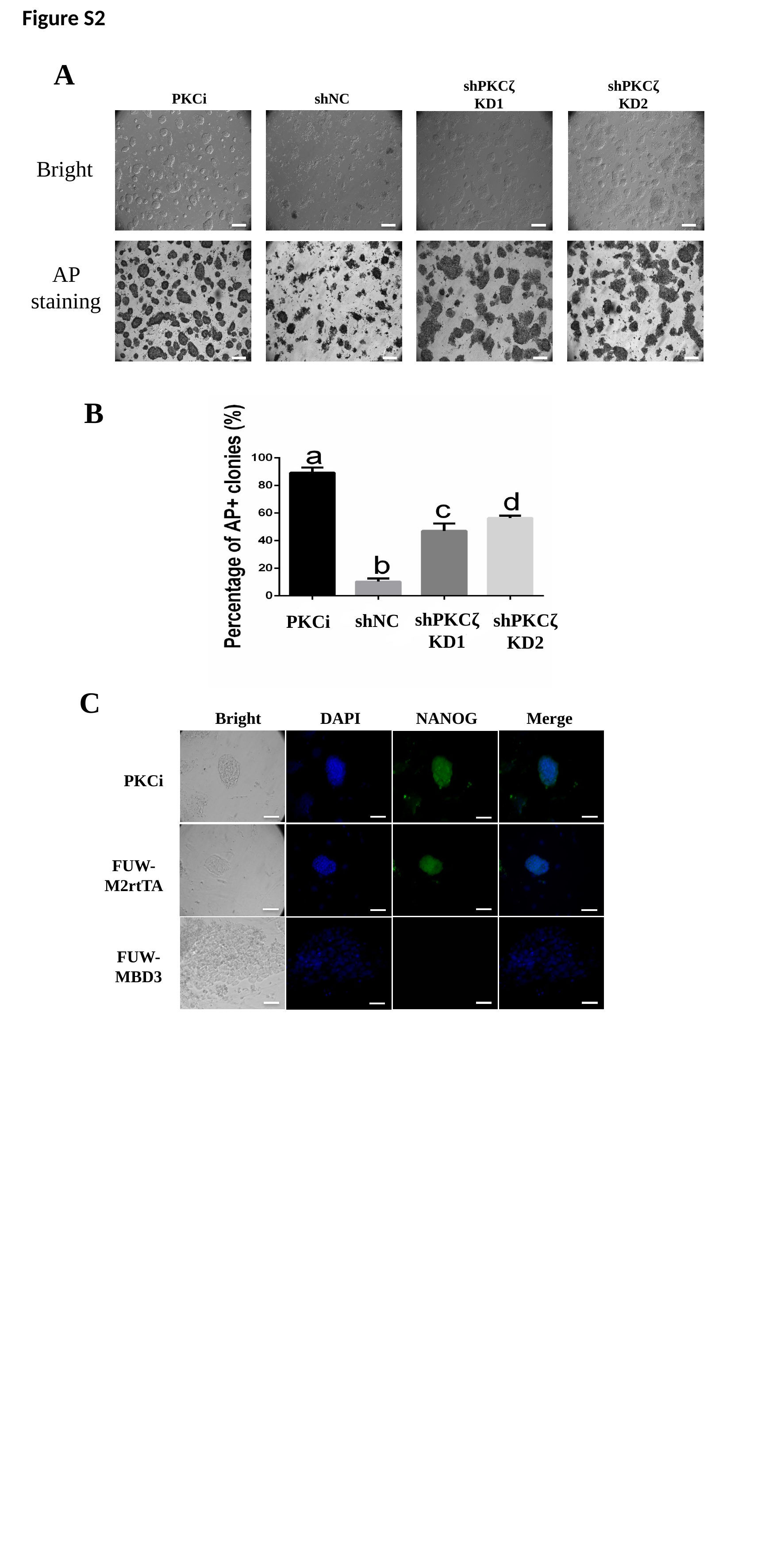

Figure S2
A
shPKCζ
KD1
shPKCζ
KD2
PKCi
shNC
Bright
AP staining
shPKCζ
KD1
shPKCζ
KD2
shNC
PKCi
C3
C4
C2
C1
B
C
Bright
DAPI
NANOG
Merge
PKCi
FUW-M2rtTA
FUW-MBD3

## Slide 3
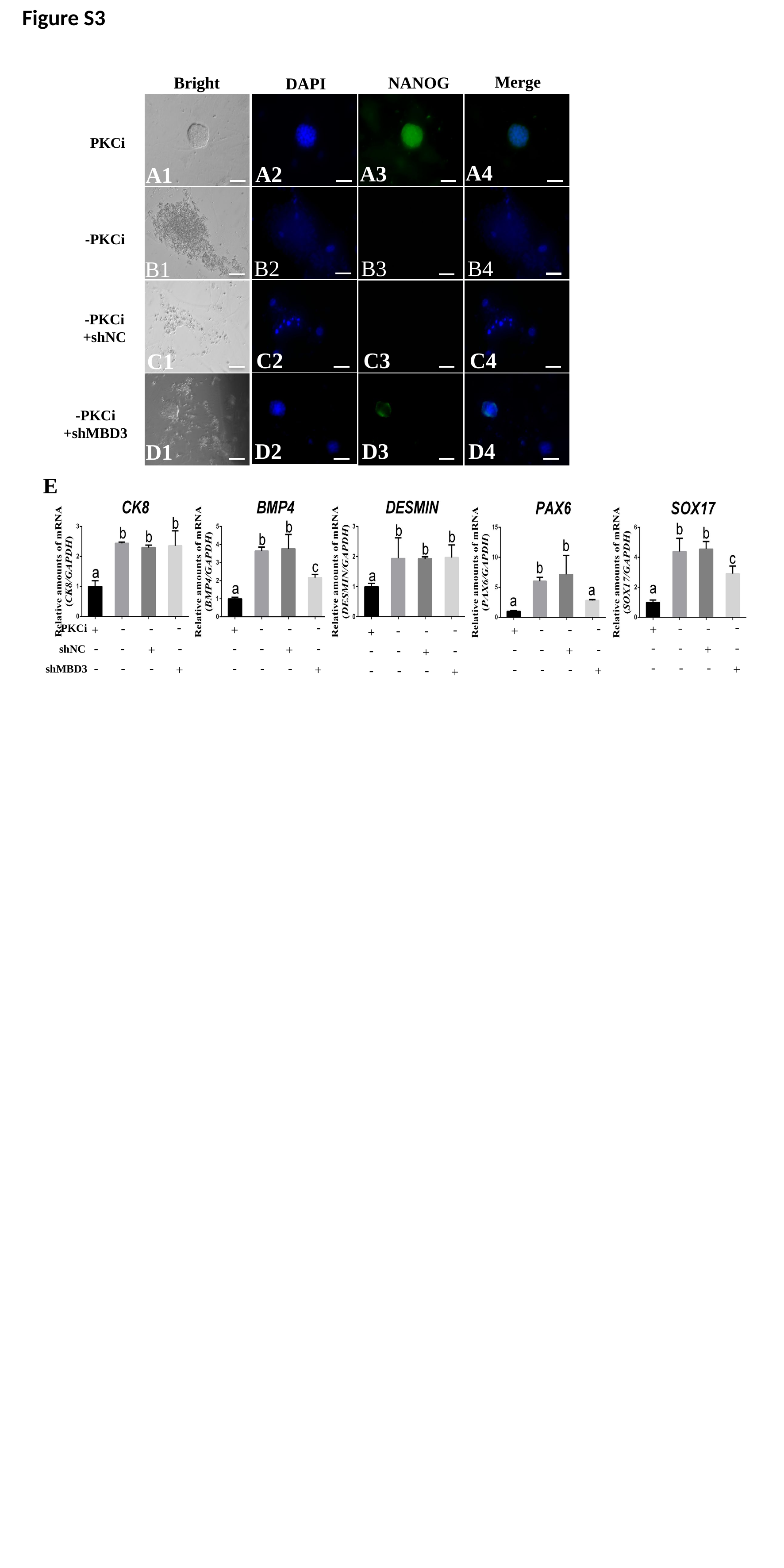

Figure S3
Merge
NANOG
Bright
DAPI
PKCi
-PKCi
-PKCi
+shNC
-PKCi
+shMBD3
A4
A3
A2
A1
B3
B4
B2
B1
C3
C4
C2
C1
D3
D4
D2
D1
E
-
-
-
-
-
+
-
-
+
+
-
-
-
-
-
PKCi
-
-
shNC
+
-
-
shMBD3
+
+
-
-
-
-
-
-
-
+
-
-
+
+
-
-
-
-
-
-
-
+
-
-
+
+
-
-
-
-
-
-
-
+
-
-
+
+
-
-
