# Supplementary material for: Nucleosome remodeling and deacetylation complex and MBD3 influence mouse embryonic stem cell naïve pluripotency under inhibition of protein kinase C
Source: Cell Death Discov. 2022 Aug 1;8:344. doi: 10.1038/s41420-022-01131-0 (PMC9343426; doi:10.1038/s41420-022-01131-0)

**Nucleosome Remodeling and Deacetylation Complex and MBD3 Influence Mouse Naïve Embryonic Stem Cell Pluripotency under Inhibition of Protein Kinase C**

**Original images in the figures**


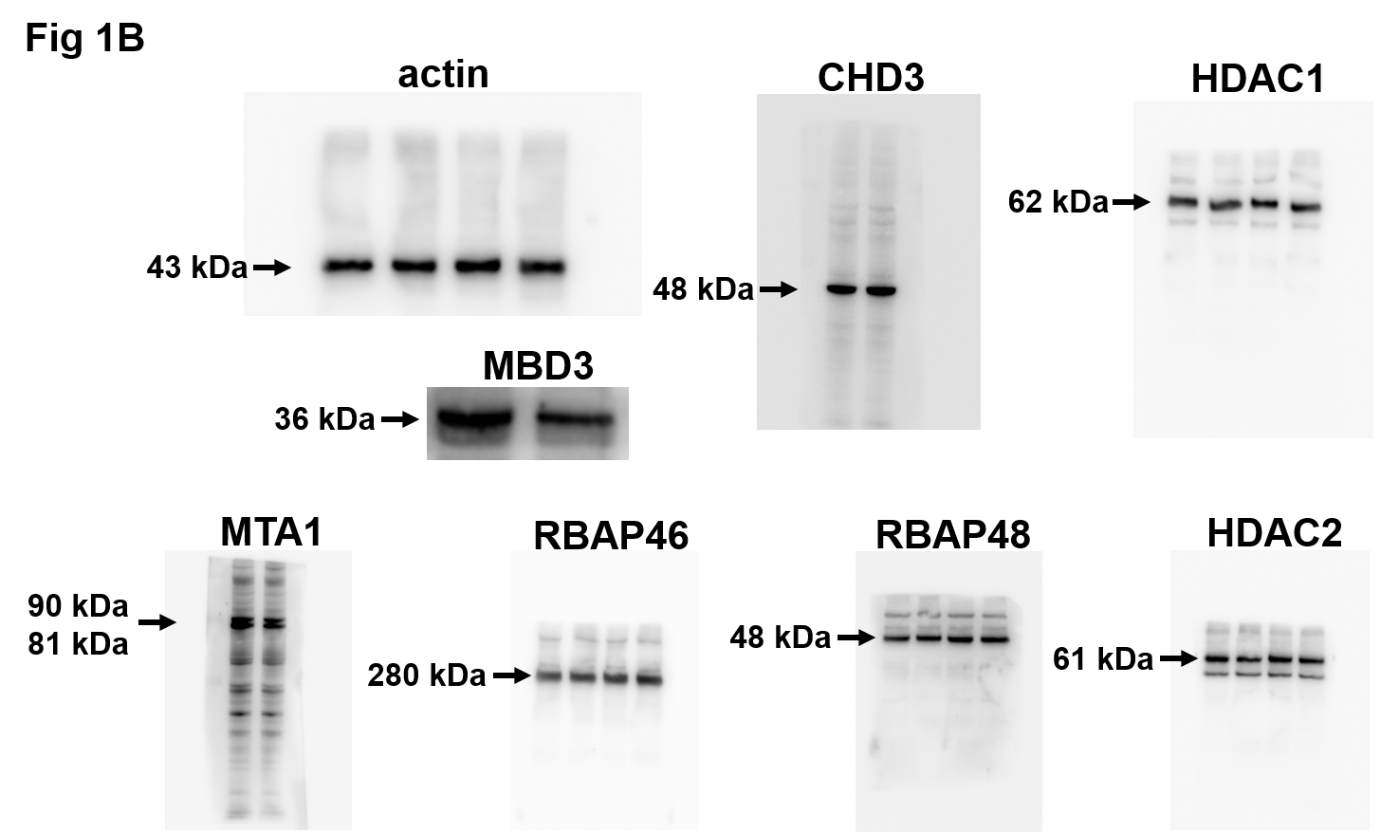


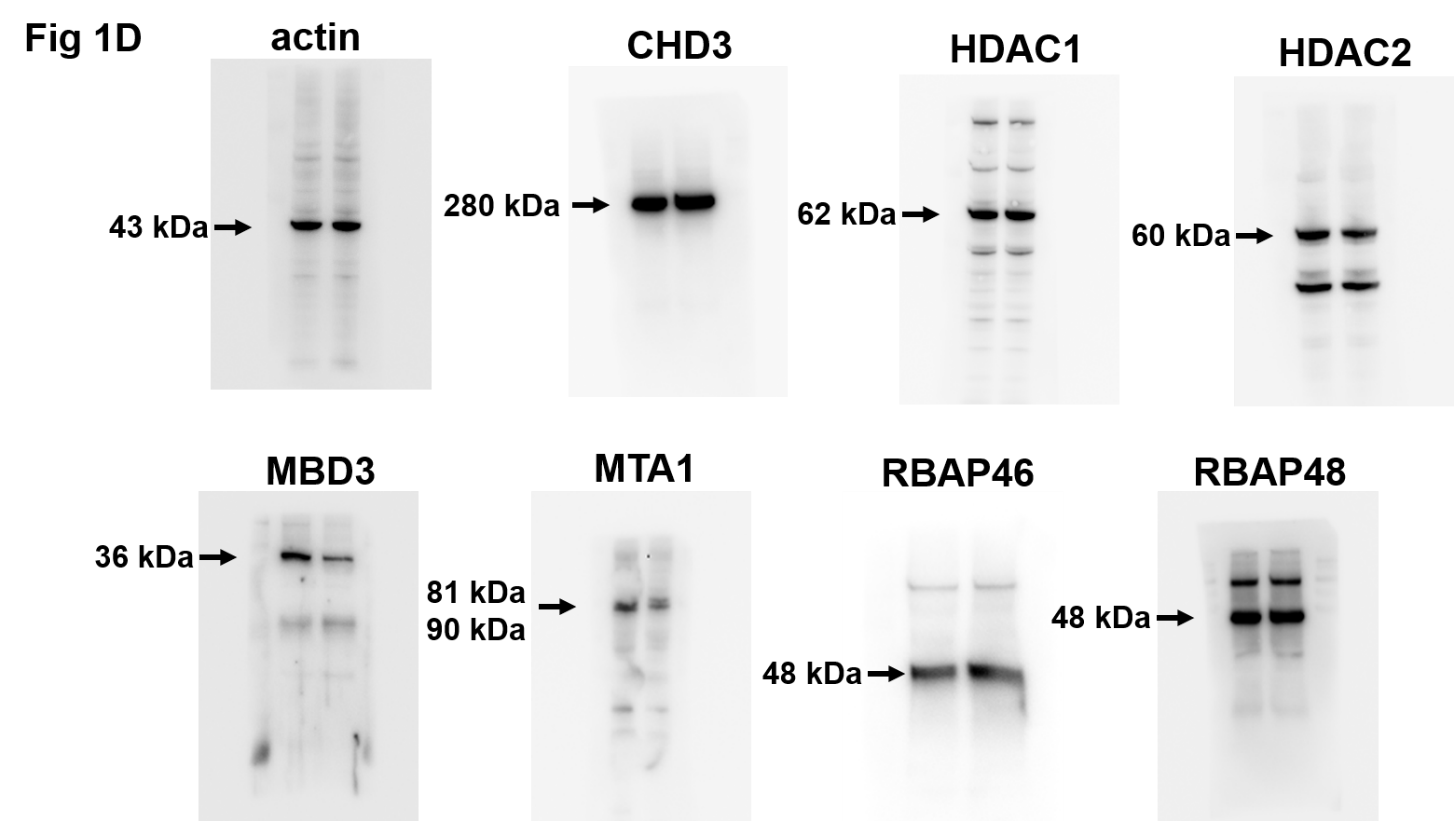


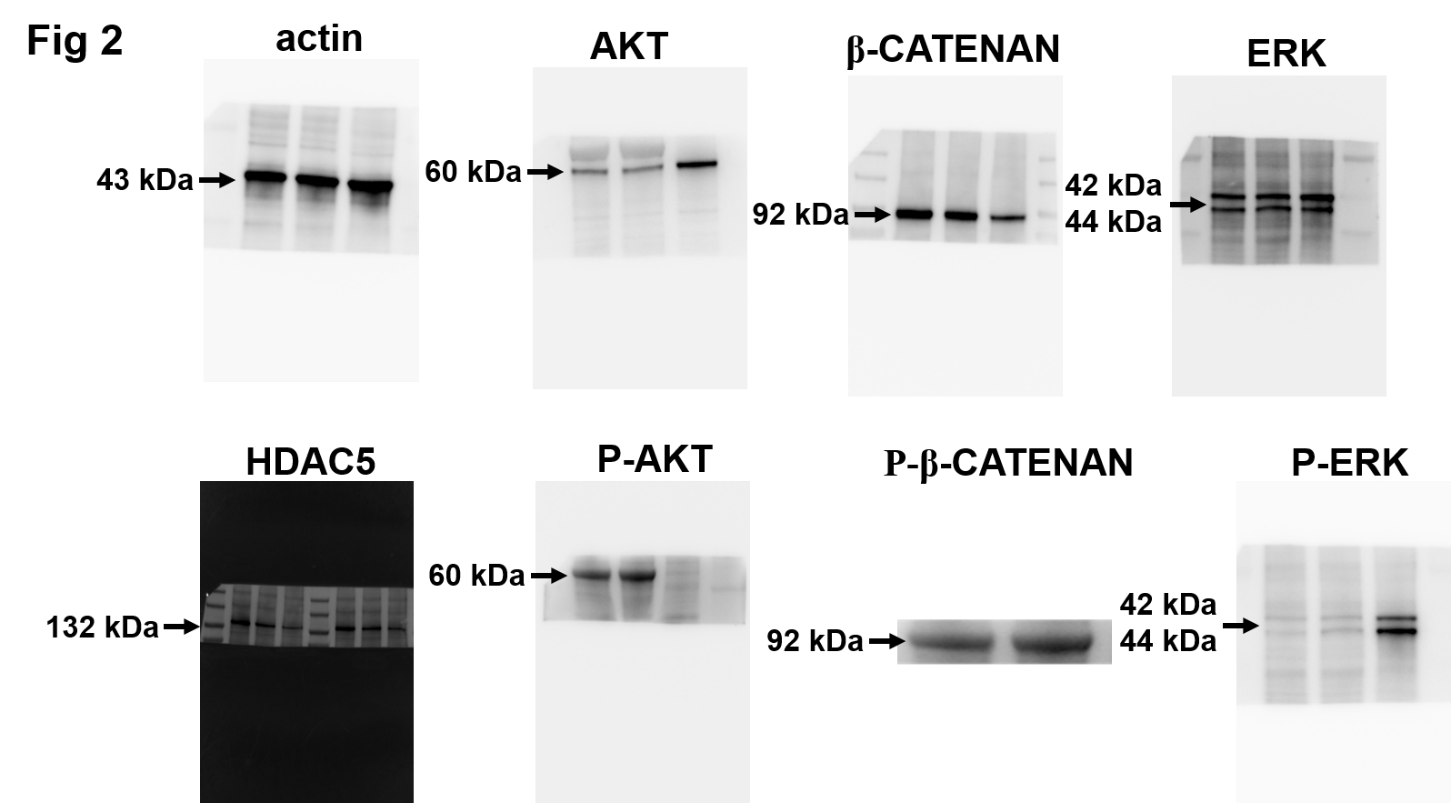


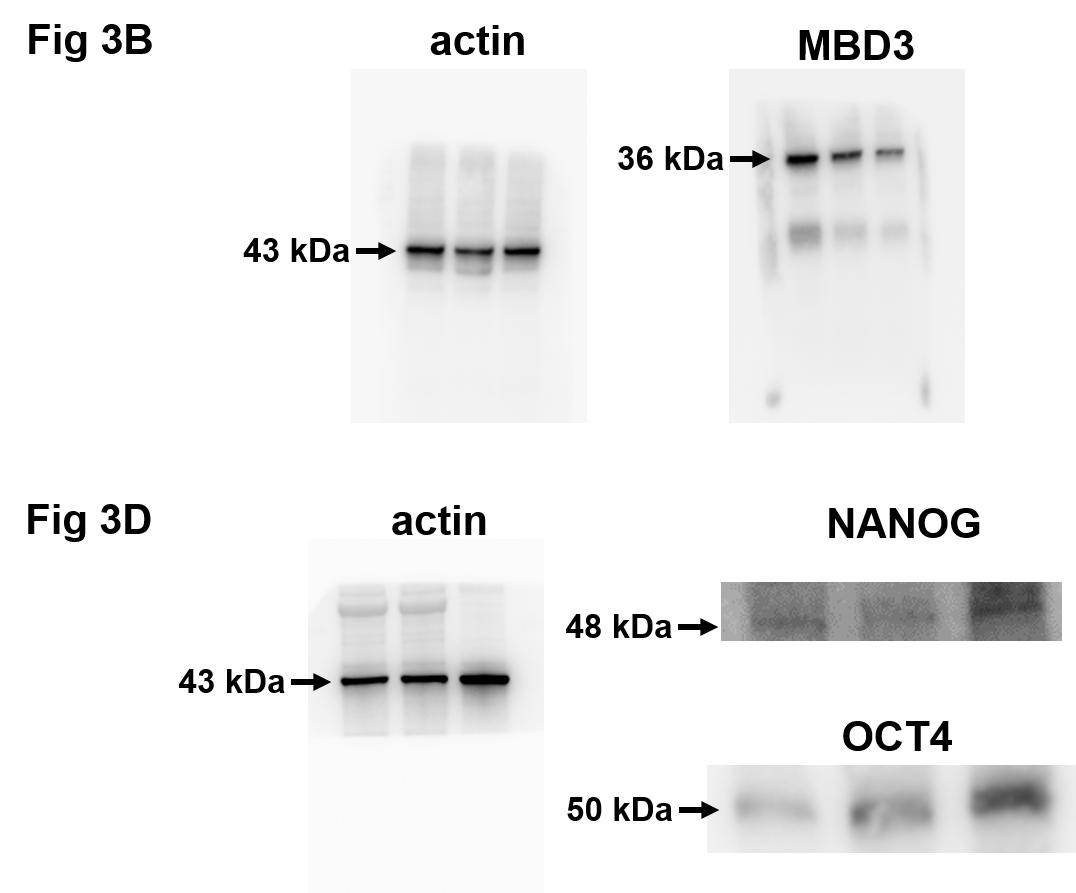


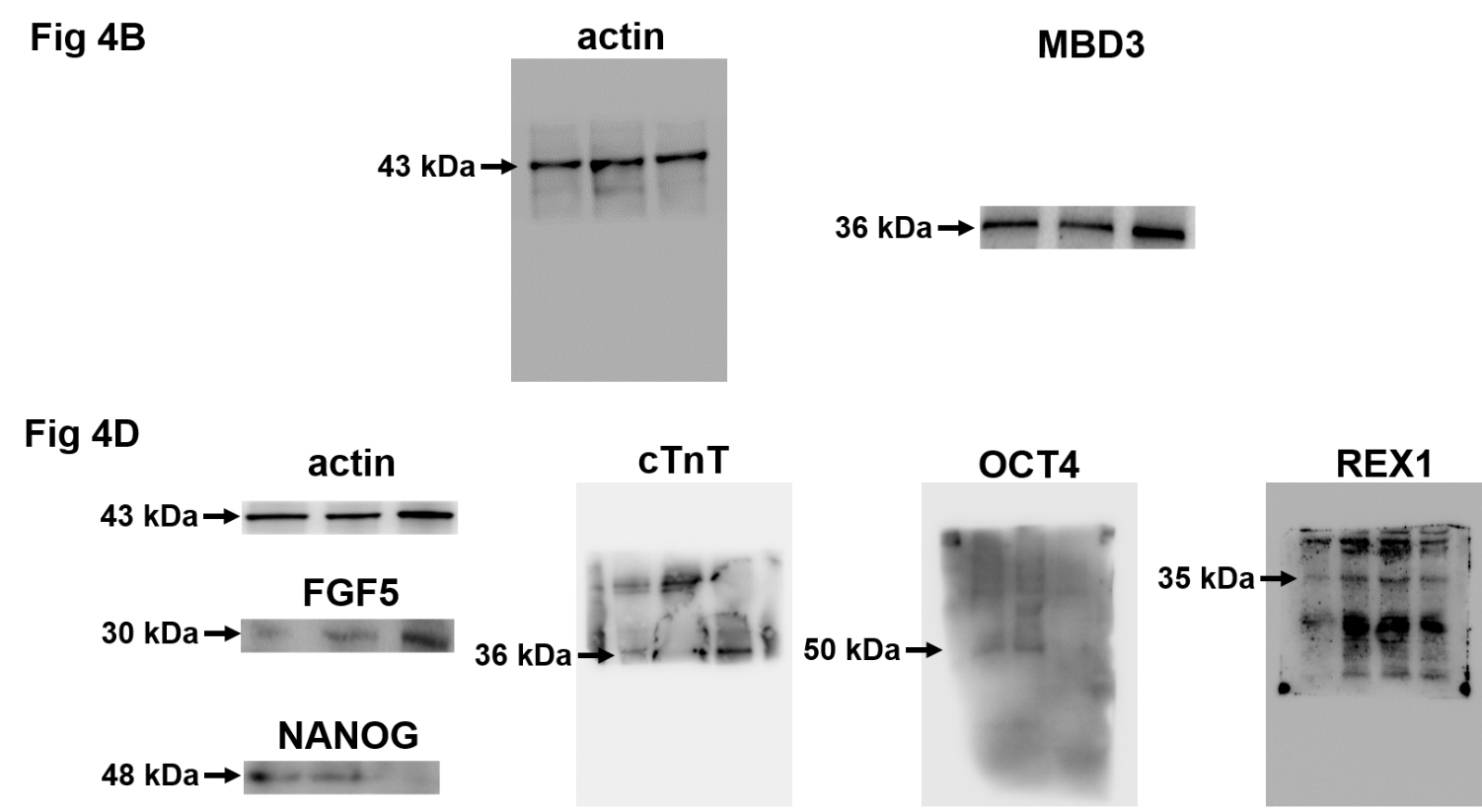


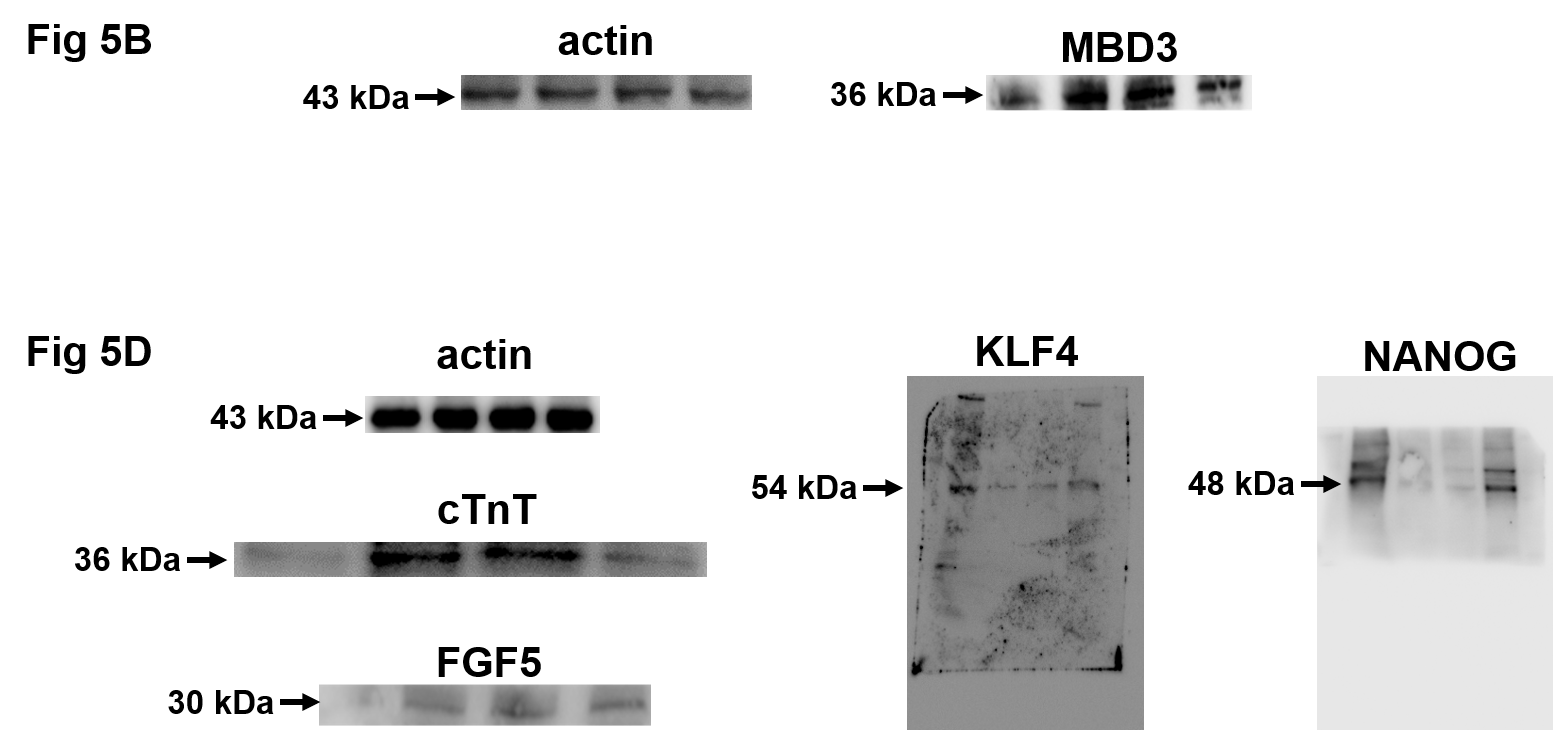

Supplement: Supplementary file 3 — Original Data File [file 41420_2022_1131_MOESM3_ESM.docx]
